# Role of nonalcoholic fatty liver disease as risk factor for drug-induced hepatotoxicity

**DOI:** 10.18053/jctres.03.2017S1.006

**Published:** 2017-02-12

**Authors:** Julie Massart, Karima Begriche, Caroline Moreau, Bernard Fromenty

**Affiliations:** 1 *Department of Molecular Medicine and Surgery, Karolinska University Hospital, Karolinska Institute, Stockholm, Sweden*; 2 *INSERM, U1241, Université de Rennes 1, Rennes, France*; 3 *Service de Biochimie et Toxicologie, CHU Pontchaillou, Rennes, France*

**Keywords:** obesity, nonalcoholic fatty liver disease, drug, liver, toxicity, drug-induced liver injury, hepatotoxicity, cytochrome P450, mitochondria, acetaminophen, halothane, isoflurane, methotrexate, tamoxifen, pentoxifylline, rosiglitazone, stavudine

## Abstract

**Background**: Obesity is often associated with nonalcoholic fatty liver disease (NAFLD), which refers to a large spectrum of hepatic lesions including fatty liver, nonalcoholic steatohepatitis (NASH) and cirrhosis. Different investigations showed or suggested that obesity and NAFLD are able to increase the risk of hepatotoxicity of different drugs. Some of these drugs could induce more frequently an acute hepatitis in obese individuals whereas others could worsen pre-existing NAFLD.

**Aim**: The main objective of the present review was to collect the available information regarding the role of NAFLD as risk factor for drug-induced hepatotoxicity. For this purpose, we performed a data-mining analysis using different queries including drug-induced liver injury (or DILI), drug-induced hepatotoxicity, fatty liver, nonalcoholic fatty liver disease (or NAFLD), steatosis and obesity. The main data from the collected articles are reported in this review and when available, some pathophysiological hypotheses are put forward.

**Relevance for patients**: Drugs that could pose a potential risk in obese patients include compounds belonging to different pharmacological classes such as acetaminophen, halothane, methotrexate, rosiglitazone, stavudine and tamoxifen. For some of these drugs, experimental investigations in obese rodents confirmed the clinical observations and unveiled different pathophysiological mechanisms which could explain why these pharmaceuticals are particularly hepatotoxic in obesity and NAFLD. Other drugs such as pentoxifyl-line, phenobarbital and omeprazole might also pose a risk but more investigations are required to determine whether this risk is significant or not. Because obese people often take several drugs for the treatment of different obesity-related diseases such as type 2 diabetes, hyperlipidemia and coronary heart disease, it is urgent to identify the main pharmaceuticals that can cause acute hepatitis on a fatty liver background or induce NAFLD worsening.

## 1. Introduction

It is currently estimated that more than 350 drugs of the modern pharmacopoeia can induce liver injury with different clinical presentations such as hepatic cytolysis and cholestasis [1,2]. Although severe drug-induced liver injury (DILI) is a rather rare event, it can require orthotopic liver transplantation, or lead to the death of the patient [2,3]. In addition, DILI can result in the withdrawal of drugs from the market, thus causing massive financial losses [4]. Thus, DILI is a major issue for public health and pharmaceutical companies.

Besides drugs characteristics such as daily dose, chemical structure and liver metabolism, different predisposing factors inherent to the patients are known to enhance the risk of DILI [1,5]. Such factors include for instance polymorphisms in genes coding for xenobiotic-metabolizing enzymes (XMEs), underlying genetic mitochondrial diseases and pre-existing liver diseases such as alcoholic liver diseases and viral hepatitis [4,6]. Another liver disease that is increasingly recognized to favor DILI is nonalcoholic fatty liver disease (NAFLD), at least for some drugs such as acetaminophen, halothane, some antiretroviral drugs and methotrexate [7,8]. The main objective of the present review is to present the available information regarding the role of NAFLD as risk factor for drug-induced hepatotoxicity. For this purpose, we performed a PubMed search of literature published in the English language using the following terms: "drug-induced liver injury", "drug-induced hepatotoxicity", "fatty liver", "nonalcoholic fatty liver disease", "steatosis" and "obesity". This data-mining analysis was also completed by using Google Scholar^®^. Before presenting the collected data concerning the present topic, some major features of NAFLD pathophysiology will be briefly recalled. Such information is important to understand why this frequent liver disease can enhance the risk of hepatotoxicity of some drugs.

## 2. Pathophysiology of NAFLD

NAFLD refers to a large spectrum of hepatic lesions linked to obesity including fatty liver, nonalcoholic steatohepatitis (NASH) and cirrhosis. NASH is characterized not only by fatty liver (also referred to as hepatic steatosis) but also by necroinflammation, some fibrosis and the presence of apoptotic hepatocytes. Importantly, most obese patients present simple fatty liver but this lesion progresses in the long term to NASH in only 10 to 20% of patients. Several genetic polymorphisms could explain, at least in part, why fatty liver progresses to NASH only in a subset of obese patients [9,10].

There is now strong evidence that insulin resistance (IR) in skeletal muscle and white adipose tissue (WAT) plays a key role in the pathogenesis of fatty liver linked to obesity [9,11]. IR in muscle is characterized by impaired glucose uptake and glycogen synthesis, thus favoring glucose utilization for hepatic de novo lipogenesis (DNL), while IR in WAT favors triacylglycerol (TAG) lipolysis. This uncontrolled lipolysis leads to the release in the circulation of large amounts of non-esterified fatty acids (NEFAs), which enter the liver in a concentration-dependent manner. In addition, NEFAs are synthesized more actively in liver because IR-associated hyperinsulinemia favors hepatic DNL. Indeed, high plasma insulin concentrations increase hepatic levels of sterol regulatory elementbinding protein 1c (SREBP1c) and peroxisome proliferator-activated receptor γ (PPARγ), two transcription factors controlling the expression of key enzymes involved in NEFA and TAG synthesis. When type 2 diabetes (T2D) occurs in obese individuals, hyperglycemia can also contributes to fatty liver by activating carbohydrate responsive element-binding protein (ChREBP), a transcription factor that enhances the expression of several glycolytic and lipogenic enzymes [9,12].

The pathogenesis of the progression of fatty liver to NASH is complex and could involve different events including overproduction of reactive oxygen species (ROS), reduced ROS detoxification, mitochondrial dysfunction, endoplasmic reticulum (ER) stress and increased release of pro-inflammatory and profibrogenic cytokines [9,11]. Some of these events could result from the direct toxicity of lipid derivatives (also referred to as lipotoxicity) on different metabolic pathways and hepatocellular constituents [9,13]. Notably, ROS overproduction during NAFLD could mainly occur within mitochondria, in particular at the level of complexes I and III of the mitochondrial respiratory chain (MRC), and of some enzymes of the fatty acid oxidation (FAO) pathway [9,14,15]. Another significant source of ROS in fatty liver could be the cytochrome P450 2E1 (CYP2E1), a XME located not only within the ER but also within mitochondria [16,17]. Thus, several mitochondrial enzymes are probably involved in ROS overproduction during NAFLD.

Numerous studies carried out in rodents and humans have reported higher hepatic CYP2E1 expression and/or activity in obesity and NAFLD [18-21]. A role of enhanced CYP2E1 activity in NAFLD pathogenesis is supported by different experimental investigations [18,22,23]. It is still unclear why CYP2E1 activity is increased in obesity and NAFLD, even though some studies suggested the role of disturbed intra-hepatic insulin signalling and/or accumulation of some endogenous derivatives such as ketone bodies and saturated fatty acids [18,20,24]. The activity of other CYPs such as CYP2C9 and CYP2D6 has also been reported to be enhanced during obesity and NAFLD [7,25]. However, higher CYP2C9 and CYP2D6 activity has most probably no significant role in NAFLD progression because these CYP isoforms do not produce ROS during their catalytic cycles, in contrast to CYP2E1 [18]. Contrary to these CYPs, hepatic CYP3A4 expression and activity are lower during obesity and NAFLD [24-26]. This might disturb the pharmacologic and safety profiles of some of the numerous drugs metabolized by this enzyme [27].

## 3. NAFLD and drug-induced hepatotoxicity

There is growing evidence that NAFLD can increase the risk and/or the severity of liver injury induced by different drugs. Practitioners should bear this information in mind because obese patients are consuming on average more drugs than non-obese individuals [7,28]. Indeed, obesity is often associated with different diseases such as T2D, hyperlipidemia, coronary heart disease and osteoarthritis that need to be treated. Moreover, obese patients can suffer from chronic diseases not necessarily related to obesity such as viral infections (i.e. HIV and HCV) and cancers. Notably, treatment of the afore-mentioned diseases requires long-term drug administration, which can enhance the risk of adverse effects including hepatotoxicity [29,30].

Obese patients are not only more likely to be exposed to different types of pharmaceuticals, but their diseased liver is also more vulnerable to some toxicological insults. Actually, hepatotoxicity in obese patients with NAFLD could occur as two distinct clinical settings [7]. For some drugs, an acute hepatitis could happen more frequently and could be more severe than for healthy patients. For some other drugs, an aggravation of the pre-existing NAFLD could occur, with for instance an accelerated transition from fatty liver to NASH.

### 3.1 Acute hepatitis in NAFLD

Numerous drugs can induce acute hepatitis [31,32], which can be sometimes severe and require the patient’s hospitalization. Indeed, acute cytolysis can involve an important proportion of hepatocytes, thus leading to hepatic failure that requires orthotopic liver transplantation [33,34]. Notably, it has been reported that subjects with drug-induced acute liver failure (ALF) were on average overweight but the body mass index (BMI) did not predict poor ALF outcome [33]. However, this study did not determine whether high BMI predicted ALF induced by some specific drugs. Actually, higher risk of acute hepatitis in obesity and NAFLD has been documented (or suspected) only with a few drugs, as listed in Table 1 and below. However, one can expect that this list will grow in the future because practitioners and researchers are increasingly aware about this issue.

**Figure 1:**
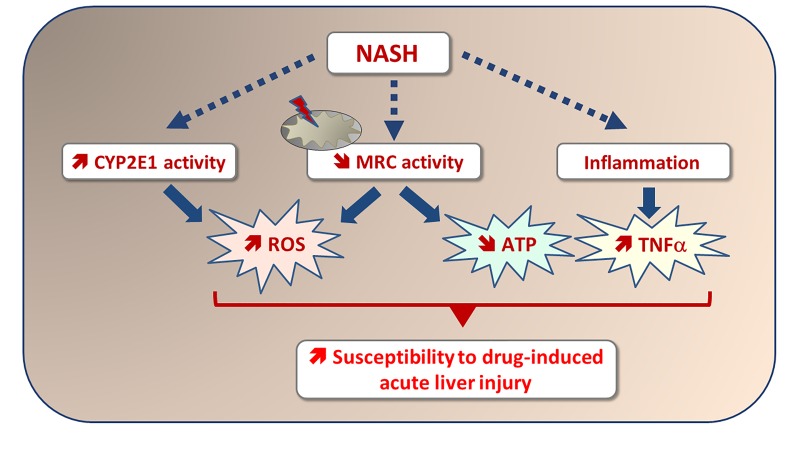
Mechanisms whereby NASH could increase the susceptibility of drug-induced acute liver injury. NASH is associated with increased CYP2E1 expression and activity, reduced MRC activity and inflammation. These events lead to ROS overproduction, reduced ATP synthesis and increased production of pro-inflammatory cytokines such as TNFα, which can favor the occurrence of drug-induced acute liver injury.

Theoretically, drug-induced acute hepatitis could occur more frequently in NAFLD because drugs are expected to be more cytotoxic in this new metabolic environment characterized by reduced ATP synthesis and higher ROS levels (Figure 1) [9,35,36]. In other words, for the same drug exposure, fatty liver will be more likely to be damaged as compared to normal liver. During NAFLD, higher ROS levels can be the consequence of both ROS overproduction by different cellular components (respiratory chain, CYP2E1 and peroxisomes) and reduced antioxidant defenses (glutathione, glutathione peroxidases, superoxide dismutases..) [9,37]. When NASH occurs, the pro-inflammatory environment with high tumor necrosis factor-α (TNFα) production could also sensitize the liver to drug-induced acute cytotoxicity (Figure 1) [38,39]. In addition to these mechanisms, drug cytotoxicity could be favored by other events such as the production of higher levels of CYP-generated toxic metabolites, as discussed below for acetaminophen and other pharmaceuticals. Finally, it must be pointed out that NAFLD might not increase the susceptibility of acute liver injury for all hepatotoxic drugs. This is most probably because the NAFLD-associated alteration of XME activities will not necessarily be associated with higher levels of the toxic compounds, which can be either the parent drugs and/or CYP-generated reactive metabolites.

**Table 1:** Drugs and acute hepatitis in obesity and nonalcoholic fatty liver disease

Drug(s)	Pharmacological class	Comments
Acetaminophen (APAP)	Analgesic and antipyretic	Several experimental and clinical studies strongly suggest that acute APAP hepatotoxicity could be more frequent and more severe in obesity and NAFLD [8,49,50,54]. This is probably because these diseases are often associated with higher hepatic CYP2E1 activity, which generates greater levels of the toxic NAPQI metabolite. However, some obese individuals do not present high CYP2E1 activity and thus they may not be at risk for APAP-induced liver injury
Amiodarone	Antiarrhythmic and antianginal	One clinical study reported that the frequency of amiodarone-induced hepatotoxicity did not appear to be greater in patients suffering from the metabolic syndrome [97]. Further investigations would be needed in order to confirm this observation
Fosinopril	Antihypertensive	Only one study reported an increased risk of fasinopril-induced hepatitis in patients with NAFLD [94]. The exact mechanism of this greater risk is currently unknown. Further investigations would be needed to confirm this clinical observation
Halothane and isoflurane	Volatile anesthetics	Several clinical studies strongly suggest that obese patients could be particularly at risk for halothane and isoflurane-induced acute hepatotoxicity [18,59-63]. This is probably because obesity is often associated with higher hepatic CYP2E1 activity, which generates greater levels of the toxic acyl chloride metabolites. However, some obese individuals do not present high CYP2E1 activity and thus they may not be at risk for halothane and isoflurane-induced liver injury
Losartan	Antihypertensive	Only one study reported an increased risk of losartan-induced hepatitis in patients with NAFLD [94]. The exact mechanism of this greater risk is currently unknown. Further investigations would be needed to confirm this clinical observation
Methotrexate	anticancer and anti-inflammatory (anti-folate)	Although several clinical studies suggest that methotrexate could induce acute hepatitis more frequently in obese patients, this drug is mostly suspected to aggravate NAFLD (see Table 2)
Omeprazole	Antiulcer (proton pump inhibitor)	Only one study reported an increased risk of omeprazole-induced hepatitis in patients with NAFLD [94]. The exact mechanism of this greater risk is currently unknown. Further investigations would be needed to confirm this clinical observation
Piperacillin/tazobactam	Antibiotics	Only one study reported an increased risk of piperacillin/tazobactam-induced hepatitis in patients with NAFLD [94]. The exact mechanism of this greater risk is currently unknown. Further investigations would be needed to confirm this clinical observation
Statins	Hypolipidemic drugs	Several clinical studies suggested that the risk of statin-induced hepatotoxicity is not higher in patients with NAFLD [95,96].
Telithromycin	Antibiotic	Only one study reported an increased risk of telithromycin-induced hepatitis in patients with NAFLD [94]. The exact mechanism of this greater risk is currently unknown. Further investigations would be needed to confirm this clinical observation
Ticlopidine	Antithrombotic (inhibitor of platelet aggregation)	Only one study reported an increased risk of ticlopidine-induced hepatitis in patients with NAFLD [94]. The exact mechanism of this greater risk is currently unknown. Further investigations would be needed to confirm this clinical observation

#### 3.1.1 Acetaminophen

In this section, we will briefly provide the most important information related to acetaminophen-induced liver injury in obesity and NAFLD, as we recently published a detailed review on the topic [8].

**Figure 2:**
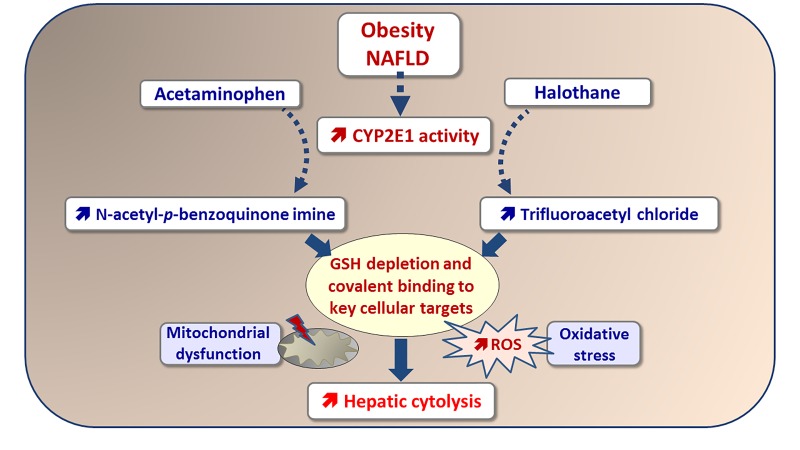
Increased hepatotoxicity induced by acetaminophen and halothane in obesity and NAFLD. Obesity and NAFLD are associated with higher hepatic cytochrome P450 2E1 (CYP2E1) activity, which is responsible for a greater biotransformation of acetaminophen and halothane to the highly reactive metabolites N-acetyl-*p*-benzoquinone imine and trifluoroacetyl chloride, respectively. These reactive metabolites bind to glutathione (GSH) and key cellular targets, in particular at the mitochondrial level. These deleterious events induce mitochondrial dysfunction, reduced ATP synthesis and oxidative stress, thus triggering hepatic cytolysis.

The painkiller acetaminophen (APAP), also known as paracetamol, is mainly metabolized in the liver into harmless glucuronide and sulfate conjugates. However, a small amount of APAP is oxidized to the highly toxic metabolite N-acetyl-*p*-benzoquinone imine (NAPQI) by several cytochromes P450, in particular CYP2E1 and CYP3A4. Hepatic CYP2E1 activity is increased not only in NAFLD, as previously mentioned, but also in other pathophysiological states including alcoholic liver disease, malnutrition and diabetes [18,40]. Thus, these diseases are expected to favor the generation of higher intrahepatic levels of NAPQI (Figure 2).

Although NAPQI is usually safely detoxified by hepatic reduced glutathione (GSH) when APAP is taken at the maximum recommended dose (i.e. 4 grams per day), high levels of NAPQI after an acute overdose consistently induce a fall in the GSH stores, thus promoting the covalent binding of free NAPQI to different cellular proteins, in particular at the mitochondrial level. This leads to profound oxidative phosphorylation (OXPHOS) impairment and ATP shortage, massive hepatocellular necrosis and ALF [8,41]. Activation of c-jun N-terminal kinase (JNK) could also be involved in APAP-induced liver injury, although some studies did not support such pathological role [24,42].

Besides the context of APAP overdose, it is noteworthy that therapeutic dose of APAP can induce mild to moderate hepatic cytolysis [21,43], and even fulminant hepatitis in a few patients [44,45]. However, it is noteworthy that no well-controlled study has reported ALF in individuals treated with recommended doses of APAP. Hence, such rare therapeutic misadventures seem to occur preferentially in patients with predisposing factors such as malnutrition, co-medication with different drugs, alcoholic liver disease and NAFLD [8,44,46]. Some of these factors can not only enhance liver CYP2E1 activity, as previously recalled, but they can also significantly reduce the intrahepatic stores of GSH [8,47,48].

NAFLD could increase the risk of APAP-induced hepatotoxicity either after an overdose or at therapeutic dosage. However, this risk might not concern all obese patients but only a subset of obese patients, as discussed below. Two large retrospective studies reported that NAFLD could be a risk factor for APAP-induced acute liver injury (ALI) after APAP overdose [49,50]. In these studies, patients with pre-existent NAFLD and hospitalized for APAP overdose had a 4-to 7-fold higher prevalence of ALI as compared to those without NAFLD [49,50]. Interestingly, these studies showed that the risk of severe ALI after APAP overdose was increased about similarly with NAFLD and alcoholic liver disease [49,50]. A recent study reported that therapeutic doses of APAP significantly increased plasma transaminases (i.e. ALT and AST) in morbidly obese patients but not in non-obese individuals [21]. Increased levels of transaminases were more than three times the reference values in some obese patients [21]. Moreover, it has been reported a case of acute fulminant hepatitis in an obese female treated by the maximal recommended dose of APAP for 10 days [44]. In contrast to these studies, the susceptibility to APAP-induced ALI was reported to be similar, or even lower, in obese patients compared to non-obese individuals [51,52]. However, one of these studies showed that obese patients had significantly poorer outcomes after ALF [51]. Although all these clinical studies are very informative, it is noteworthy that some confounding factors were not taken into account, such as different psychological and genetic factors.

Several studies in obese rodents showed that obesity and NAFLD are actually not sufficient by themselves to favor APAP-induced hepatotoxicity [8,53,54]. In contrast, our investigations in ob/ob and db/db obese mice clearly indicated that greater APAP hepatotoxicity was correlated with higher hepatic CYP2E1 activity but not with the importance of fat accumulation in liver [54]. Thus, taking into account these data and other studies, we recently proposed that the occurrence of APAP-induced hepatotoxicity in an obese individual will depend on a subtle balance between metabolic factors that can be protective (i.e. lower absorption rate, higher volume of distribution, reduced CYP3A4 activity and increased APAP glucuronidation in liver) and others that can enhance the hepatic production of NAPQI (CYP2E1 induction) or lessen the detoxification rate of this toxic metabolite (lower GSH stores) [8].

The high variability of hepatic CYP2E1 activity in obese individuals could also explain why all these subjects are apparently not at risk for APAP-induced hepatotoxicity. Although different clinical investigations consistently showed that liver CYP2E1 activity is statistically greater in obesity and NAFLD, this activity is highly variable in obese patients [21,55-58]. In particular, whereas some obese patients present very high hepatic CYP2E1 activity, others are in the range of non-obese individuals [21,55-58]. Further investigations would be needed to determine whether some simple biomarkers could predict high hepatic CYP2E1 activity in obese individuals. Indeed, such biomarkers might be interesting to identify the patients for whom APAP should be avoided, in particular in long-term treatment. Finally, variability in hepatic GSH levels might also exist in obese individuals. Indeed, GSH stores could be lower in patients with NASH, as compared to individuals with simple fatty liver, because NASH is consistently associated with higher oxidative stress [8,9]. Consequently, greater oxidative stress and lower liver GSH at baseline could increase the threshold of APAP toxicity in certain obese patients [8].

#### 3.1.2 Halothane and isoflurane

Halothane and isoflurane are two volatile halogenated anesthetics that have been reported to induce ALI more frequently in obese individuals [18,59-63]. Moreover, obesity seems to be associated with poor prognosis during halothane and isoflurane-induced hepatitis [60,61]. However, it is noteworthy that the above-mentioned investigations were based on relatively small number of patients and thus larger studies would be needed. Besides obesity, other factors such as female gender and repeated exposures to these halogenated anesthetics could also enhance the risk and severity of hepatotoxicity [61,63,64]. In the most severe cases, halothane and isoflurane are able to induce massive liver necrosis and fulminant hepatic failure that can require orthotopic liver transplantation, or even lead to the death of the patients [59,61,63]. These anesthetics can also induce cholestasis and steatosis in some patients [31,59,61].

Both halothane and isoflurane are predominantly oxidized in the liver by CYP2E1. Importantly, this CYP2E1-mediated oxidation generates electrophilic acyl chloride reactive metabolites able to covalently bind to a large array of cellular proteins [18,65,66]. Regarding halothane, covalent binding of the trifluoroacetyl chloride metabolite to key cellular targets is deemed to be involved in liver injury by triggering an immune-mediated reaction but also by inducing mitochondrial dysfunction, GSH depletion and oxidative stress [67-71]. Similar mechanisms of hepatotoxicity have been proposed for isoflurane [65,72]. Thus, taking into account the key role of CYP2E1 in the pathogenesis of halogenated anesthetic-induced hepatotoxicity, it is likely that the greater risk of liver injury in obese patients treated with halothane or isoflurane might be the consequence of the higher CYP2E1 activity commonly observed in obese patients (Figure 2) [7,18]. In keeping with this assumption, plasma levels of trifluoroacetic acid (i.e. a metabolite generated from trifluoroacetyl chloride) tended to be higher in morbidly obese patients compared to lean individuals [73]. Another study performed in morbidly obese individuals reported higher halothane biotransformation to the ionic fluoride metabolite [74]. Unfortunately, unlike the painkiller APAP, no investigations have been carried out in experimental NAFLD models in order to confirm that higher hepatic CYP2E1 activity is responsible for increased halothane hepatotoxicity in obesity. Finally, it should be pointed out that the higher risk of halothane and isoflurane-induced hepatotoxicity might not concern all obese patients but only a subset of patients who present the highest hepatic CYP2E1 activity, as previously discussed for APAP.

#### 3.1.3 Methotrexate

Methotrexate (MTX) is a folate antagonist used in the treatment of some malignancies and different autoimmune and inflammatory diseases such as psoriasis, psoriatic arthritis and rheumatoid arthritis. Supplementation with folic acid (or folinic acid) is recommended in treated patients in order to reduce the risk of MTX-induced adverse effects including hepatotoxicity [75,76]. Notably, MTX-induced liver toxicity is mostly associated with its long-term use for the treatment of inflammatory diseases [77,78]. Although some cases of acute hepatitis with marked increase in plasma transaminases and hepatocellular necrosis have been reported in a few patients treated with high doses of intravenous MTX [79,80], this drug mostly induces chronic forms of liver lesion such as steatosis, steatohepatitis, fibrosis and even cirrhosis [31,78,80,81-83]. Psoriatic patients appear to have a higher risk for MTX-induced liver fibrosis. Indeed, several studies reported the presence of hepatic fibrosis in at least 20% of the treated psoriatic patients, although a majority of these cases corresponded to mild fibrosis [77,84,85].

Different studies reported that obesity and T2D could increase the risk of MTX-induced hepatotoxicity as assessed by increased plasma transaminases, or the presence of hepatic alterations such as steatohepatitis, fibrosis or cirrhosis [81,85,86-90]. However, when considering the investigations reporting only plasma transaminases [87,90], it is unclear whether MTX-induced transaminase elevation was actually linked to the occurrence of an acute hepatitis or a chronic form of liver injury such as steatohepatitis, or alternatively, to the worsening of pre-existing NAFLD. Because MTX-induced acute hepatitis seems to be an uncommon adverse event, it is likely that most cases of transaminase elevation during MTX treatment in obese and diabetic patients might be linked to the occurrence of a chronic form of liver injury, or to an aggravation of NAFLD. Hence, the issue of MTX-induced aggravation of pre-existing NAFLD and its possible pathophysiology will be discussed below. Finally, it is noteworthy that other factors seem to increase the risk of MTX-induced hepatotoxicity including cumulative dose and duration of MTX therapy, lack of folate supplementation, hyperlipidemia, alcohol abuse and genetic factors [87,90-93].

#### 3.1.4 Other drugs

In a 6-year prospective study carried out in 74 patients with NAFLD and 174 with HCV-related chronic hepatitis, it has been found that NAFLD was an independent risk of DILI with an odds ratio of almost 4 [94]. In this study, ALI was observed in obese patients treated with fosinopril and losartan (antihypertensive agents), piperacillin/tazobactam and telithromycin (antibiotics), omeprazole (proton pump inhibitor) and ticlopidine (inhibitor of platelet aggregation) [94]. Although this report is interesting, prospective studies on larger population are needed in order to determine whether NAFLD is a *bona fide* independent risk of hepatotoxicity induced by these drugs.

Other investigations suggested that some drugs might not induce more frequently acute hepatitis in obese patients with NAFLD. These drugs include statins [95,96] and amiodarone [97]. However, these investigations were retrospective and further studies are warranted to confirm the innocuousness of these drugs in obese patients. Nonetheless, these reports suggest that obesity and NAFLD might not increase the risk of acute hepatitis for all hepatotoxic drugs, as previously mentioned.

### 3.2 Aggravation of pre-existing NAFLD

Clinical and experimental investigations indicate that chronic administration of some drugs can aggravate pre-existing NAFLD. Actually, the fact that NAFLD can be aggravated by long-term exposure to some xenobiotics is not restricted to drugs. For instance, different experimental and clinical studies have consistently shown that chronic alcohol abuse is particularly harmful for the steatotic liver and can severely worsen NAFLD [98-103].

**Figure 3:**
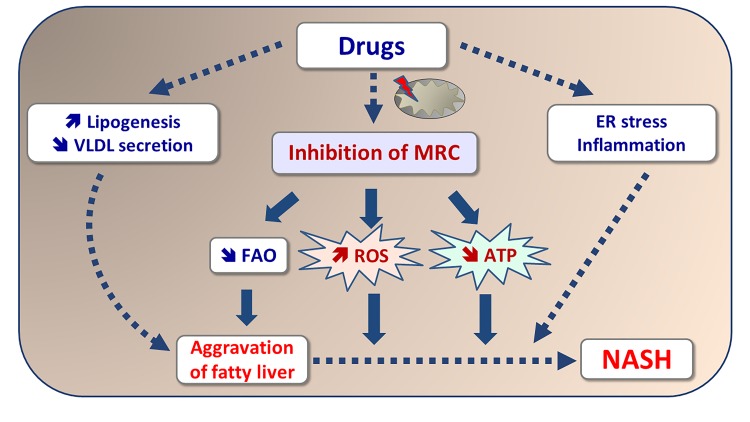
Mechanisms whereby some drugs could aggravate fatty liver and promote its progression to NASH in obese patients. Drug-induced worsening of fatty liver could be secondary to reduced VLDL secretion, increased lipogenesis and impaired mitochondrial FAO. The progression of fatty liver to NASH could be triggered by lower ATP production, higher mitochondrial ROS production, ER stress and inflammation. Notably, drug-induced inhibition of MRC could be a common mechanism leading to impaired FAO, reduced ATP levels and higher ROS production.

As previously mentioned, NAFLD refers to a large spectrum of hepatic lesions including fatty liver, NASH and cirrhosis. Hence, aggravation of NAFLD can be secondary to an exacerbation of the intrahepatic accumulation of fat and/or a faster progression of the disease, for instance through a quicker transition from fatty liver to NASH. Conceptually, xenobiotics could exacerbate hepatic fat accretion by increasing DNL, limiting very low-density lipoprotein (VLDL) excretion and/or reducing mitochondrial FAO (Figure 3) [7,104]. Furthermore, a more rapid transition towards NASH could primarily involve ROS overproduction by the dysfunctional MRC, ER stress, lower antioxidant defences and higher expression of pro-inflammatory cytokines such as TNFα (Figure 3) [6,7]. Interestingly, almost all the above-mentioned events seem to be involved in NAFLD aggravation induced by chronic ethanol intoxication [101-103].

Actually, impairment of the MRC activity could be a central mechanism whereby some xenobiotics including drugs are able to worsen NAFLD (Figure 3) [6,7]. Indeed, MRC inhibition could not only contribute to the aggravation of fatty liver *via* the secondary impairment of mitochondrial FAO but also to the occurrence of necrosis secondary to ATP deficiency. In addition, MRC impairment is leading to higher mitochondrial ROS production, which could favor hepatic fibrosis [105,106]. Interestingly, several drugs discussed in the following sections were reported to inhibit the MRC activity. Table 2 summarizes the main data collected for these drugs.

**Table 2:** Drugs and aggravation of pre-existing NAFLD

Drug(s)	Pharmacological class	Comments
Acetaminophen (APAP)	Analgesic and antipyretic	Some experimental studies in rodents suggest that chronic treatment with therapeutic doses of APAP could have deleterious effects on liver function and carbohydrate homeostasis [113,115,116]. Although it is currently unclear whether fatty liver might be particularly affected by long-term APAP treatment, further investigations are warranted in order to determine whether APAP can be safely prescribed for prolonged periods in obese people
Corticosteroids	Anti-inflammatory	Experimental studies showed that corticosteroids aggravated fatty liver in obese rodents [121,122]. Some experimental data also suggested that these drugs could also trigger NASH [121]. Hence, corticosteroids should be used with caution in obese patients
Anticancer	Irinotecan	Clinical studies reported that irinotecan administration before liver resection could induce steatohepatitis more frequently in obese individuals [126,127]
Methotrexate (MTX)	anticancer and anti-inflammatory (anti-folate)	Several clinical investigations suggested that MTX might aggravate NAFLD, in particular hepatic fibrosis [81,129,130]
Pentoxifylline (PTX)	Hemorrheologic agent with anti-TNF properties	PTX might aggravate NAFLD in a few patients [166,167]. Investigations in obese mice suggested that PTX-induced worsening of fatty liver could be secondary to hyperglycemia and higher hepatic DNL [171]
Phenobarbital	Antiepileptic	Experimental investigations showed that phenobarbital exacerbated hepatic lipid deposition in obese rats [180]. No data are available for obese patients
Raloxifene	Anti-osteoporotic (selective estrogen receptor modulator)	A case report described a case of NASH aggravation in a female patient treated with raloxifene [215]. Further investigations would be needed in order to determine whether raloxifene could pose a potential risk in obese patients
Rosiglitazone	Antidiabetic (PPARγ agonist)	Worsening of necroinflammation, perisinusoidal fibrosis and steatosis was observed in some obese patients treated with rosiglitazone [195,197,198]. Experimental investigations performed in different murine models of obesity and T2D confirmed that rosiglitazone was able to exacerbate hepatic steatosis and liver TAG accumulation [200-205]. Some experimental data also suggested that necroinflammation could also be worsened by rosiglitazone [200,202]
Stavudine (d4T)	Antiretroviral (NRTI)	Some clinical investigations suggested that d4T could induce fatty liver disease or worsen NAFLD in some patients, although further investigations would be needed to ascertain NAFLD aggravation [119,154-156]. Other studies also suggested that the risk of d4T-induced lactic acidosis could be higher in overweight or obese female patients [159-162]. Other NRTIs such as didanosine (ddI) might also aggravate NAFLD in obese patients [156].
Tamoxifen	Anticancer (selective estrogen receptor modulator)	Some clinical studies showed that obesity enhanced the risk of tamoxifen-induced steatohepatitis [211,213]. However, further investigations are needed in order to determine whether tamoxifen is able to worsen pre-existing NAFLD in obese patients
Tetracycline	Antibiotic	Experimental investigations showed that tetracycline exacerbated hepatic TAG deposition and triggered steatohepatitis in obese mice [231]. However, this interesting observation is not clinically relevant because tetracycline is no longer prescribed in patients

#### 3.2.1 Acetaminophen (APAP)

As previously mentioned, therapeutic doses of APAP can induce liver injury in some patients. About one third of treated patients could have a significant increase in their plasma transaminases after 1 or 2 weeks of APAP treatment [43,107]. Although extremely rare, fulminant hepatitis have been reported in patients after several days of APAP treatment at recommended dosage [44,45]. Moreover, long-term intake of APAP has been reported to induce chronic liver injury in a few treated patients, such as cirrhosis and granulomatous hepatitis [31,108]. Interestingly, some data suggest that chronic APAP ingestion could significantly reduce GSH stores, at least in some treated patients [109,110]. Lastly, therapeutic doses of APAP could also have detrimental effects on the kidney and the cardiovascular system [111,112]. Thus, therapeutic doses of APAP might be at risk for some individuals, in particular during chronic treatment. Accordingly, several investigations in rodents showed that long-term treatment with therapeutic doses of APAP was able to induce oxidative stress and cellular injury in different tissues [113-115]. Interestingly, a single low dose of APAP in mice induced reversible mitochondrial dysfunction and steatosis in liver, but this study did not determine the effects of repeated administration of such low APAP dose [116].

There is currently no information as to whether NAFLD might be aggravated in some patients by therapeutic doses of APAP. However, recent experimental studies performed in our laboratory suggested that APAP administration might not be safe in NAFLD (Begriche et al., manuscript in preparation). Indeed, a 1-month treatment with therapeutic doses of APAP significantly enhanced plasma aspartate aminotransferase (AST) activity by 45% in mice fed a high-fat diet (HFD), whereas this increase was only 19% in mice fed a normal diet. Furthermore, HFD obese mice treated by APAP presented a higher hepatic mRNA expression of several proteins responsive to cellular stress including γ-glutamylcysteine synthetase (γ-GCS), tribbles pseudokinase 3 (Trib3) and X-box binding protein 1 (Xbp1). However, chronic APAP treatment concomitantly induced in obese mice a significant reduction of liver TAG levels associated with lower plasma insulin and reduced liver expression of different genes involved in DNL including SREBP1c, stearoyl-coenzyme A desaturase 1 (SCD1) and glycerol-3-phosphate acyltransferase (GPAT). In contrast to insulin, blood glucose was not significantly changed by APAP. Other studies in mice reported the effect of chronic treatment with therapeutic doses of APAP on glucose homeostasis and insulin resistance. In two studies from the same group, APAP was reported to alleviate impaired glucose intolerance and hyperinsulinemia in HFD obese mice [117,118]. However, long-term administration of APAP induced glucose intolerance and hepatic insulin resistance in mice fed a chow diet [115]. Furthermore, APAP decreased serum insulin and β-cell relative volume in these mice [115]. Clearly, further investigations are warranted in order to determine whether APAP can be safely prescribed for chronic treatment, in particular in patients suffering from obesity and related metabolic disorders.

#### 3.2.2 Corticosteroids

These drugs are used in numerous diseases for their antiinflammatory and anti-allergic effects. However, one of the main adverse effects of this drug class is the occurrence of metabolic and endocrine disturbances and in particular obesity, hyperlipidemia, steatosis, steatohepatitis, IR and diabetes [6,7,119,120]. In two independent studies carried out in HFD obese rodents, long-term treatment with corticosteroids (corticosterone or dexamethasone) significantly worsened hepatic steatosis [121,122]. In one of these studies, plasma ALT, total bilirubin and liver collagen content were significantly enhanced in HFD obese rats treated with corticosterone whereas no such disturbances were observed in untreated HFD rats [121]. Thus, in the context of obesity, corticosteroids might not only aggravate fatty liver but might also trigger NASH. Interestingly, corticosteroids are able to disturb mitochondrial FAO, MRC activity and oxidative phosphorylation in liver [123-125]. However, further investigations would be required to determine whether mitochondrial dysfunction is involved in NAFLD worsening in corticosteroid-treated rodents [121,122].

#### 3.2.3 Irinotecan

This topoisomerase 1 inhibitor, also known as CPT-11, is mainly used for the treatment of advanced colorectal cancer. Irinotecan is also used before resection of hepatic colorectal metastases. Two independent clinical studies reported that irinotecan administration before liver resection could induce steatohepatitis (sometimes severe) in treated patients and that the risk of liver injury appeared to be greater in obese individuals [126,127]. Although the reason of this increased susceptibility is currently unknown, recent experimental investigations in mice showed that obesity was associated with higher plasma levels of the toxic irinotecan metabolite, SN-38 [128].

#### 3.2.4 Methotrexate

As previously mentioned, it is likely that most cases of transaminase elevation in obese and diabetic patients treated with MTX could be linked to the occurrence of a chronic form of liver injury such as steatohepatitis, or to an aggravation of pre-existing NAFLD. Although this issue is still unclear, an aggravation of NAFLD is supported by a clinical study carried out in a small group (n=20) of obese and/or diabetic patients for whom serial liver biopsies were performed [81]. In this study, fibrosis was aggravated in three-quarter of the treated patients, whereas steatosis and necroinflammation were worsened in the remaining individuals [81]. Thus, although these data indicated that all histological components of NAFLD could be worsened by MTX, it seems that this drug could more frequently aggravate hepatic fibrosis. It is noteworthy that other studies using non-invasive methods such as ultrasound elastography also suggested that MTX might aggravate liver fibrosis in obese patients [129,130]. However, a serial assessment of hepatic fibrosis was not performed in these studies. Interestingly, recent investigations in a rat model of steatohepatitis induced by a methionine choline-deficient (MCD) diet showed that a single injection of MTX was able to induce liver fibrosis in the MCD rats whereas no fibrosis was observed in rats fed with a control diet [131,132]. Although these data are interesting, it should be underlined that the MCD model of NASH does not reflect the metabolic context of obesity because the MCD diet induces a significant loss of body weight and lower glycemia [9,133].

Although the exact mechanism whereby MTX could aggravate NASH is still unknown, one attractive hypothesis is mitochondrial dysfunction (Figure 3). Indeed, MTX-induced mitochondrial dysfunction has been shown in different experimental models [104,134,135]. In particular, our recent investigations in isolated mouse liver mitochondria showed that relatively low concentrations of MTX (~40-50 μM) were able to inhibit oxygen consumption with glutamate/malate or palmitoyl-L-carnitine/malate as respiratory substrates [104]. These mitochondrial effects might explain why MTX is able to induce hepatic steatosis, necrosis and fibrosis, as already discussed.

A previous study in patients with MTX-induced liver fibrosis reported a hepatic overexpression of different genes encoding complement components including C3, C5 and C8a [136]. Interestingly, activation of the complement system could play a role in the progression of NAFLD [137,138] and in the occurrence of fibrosis in other liver diseases [139,140]. Thus, MTX might aggravate NAFLD and especially fibrosis by further activating the complement system in liver. Although MTX-induced mitochondrial dysfunction and complement activation might be attractive hypotheses, it is clear that further investigations are needed to decipher the mechanism(s) whereby MTX is able to worsen NAFLD in obese patients.

#### 3.2.5 Nucleoside reverse transcriptase inhibitors

The nucleoside reverse transcriptase inhibitors (NRTIs) are efficient drugs used for the treatment of HIV infection. NRTIs include abacavir (ABC), didanosine (ddI), lamivudine (3TC), stavudine (d4T) and zidovudine (AZT). These drugs can cause various adverse effects, sometimes severe and fatal, including lactic acidosis, bone marrow suppression, myopathy, pancreatitis and liver injury [141-143]. NRTI-induced hepatotoxicity includes hepatic cytolysis, microvesicular and macrovacuolar steatosis, steatohepatitis and cirrhosis [104,141-143]. This hepatic toxicity is particularly observed with d4T, ddI and AZT [31,143]. These latter NRTIs can also induce lipoatrophy, which is characterized by a loss of WAT in different body areas [141,144,145]. Notably, NRTI-induced lipoatrophy could promote secondary IR in some tissues including WAT and muscle [146,147]. It is acknowledged that most NRTI-induced adverse effects are caused by depletion of mitochondrial DNA (mtDNA), a small genome encoding for 13 proteins mandatory for MRC activity and OXPHOS [6,141,143]. Importantly, mtDNA depletion-induced MRC alteration in turn leads to an impairment of mitochondrial FAO and tricarboxylic acid (TCA) cycle, responsible for hepatic steatosis and lactic acidosis, respectively [6,141,148-150]. However, it is noteworthy that NRTI-induced toxic effects not related to mtDNA depletion were also reported in different experimental investigations [141,151-153].

Some NRTIs, in particular d4T and ddI, are suspected to induce fatty liver disease or worsen NAFLD in treated patients [119,154-156]. Several different mechanisms, which are not mutually exclusive, might be involved in these deleterious effects. First, NRTI-induced impairment of hepatic MRC activity could be involved, as previously discussed (Figure 3). Second, NRTIs could also induce hepatic lipid deposition by inducing ER stress and inhibiting autophagy activity [157,158]. Third, NRTI could promote fatty liver by way of lipoatrophy-related IR [154,155], in a similar way than for obesity-induced IR [9,11].

It is noteworthy that several studies suggested that the risk of d4T-induced lactic acidosis could be higher in overweight or obese female patients [159-162]. Moreover, a high BMI might increase the risk of fatal lactic acidosis [163]. Although the exact mechanisms underlying this higher risk are currently unknown, it is conceivable that d4T treatment might have significantly exacerbated NAFLD-associated mitochondrial dysfunction in these obese individuals, thus triggering severe TCA cycle deficiency and lactic acidosis. Unfortunately, these investigations did not determine whether d4T also worsen fatty liver or NASH in these patients.

#### 3.2.6 Pentoxifylline

The methylxanthine derivative pentoxifylline (PTX) is a nonselective phosphodiesterase inhibitor commonly prescribed for peripheral vascular disorders and intermittent claudication by improving blood flow and circulation. Because PTX is also an inhibitor of TNFα synthesis, it has also been tested in different diseased states involving this pro-inflammatory cytokine such as alcoholic hepatitis and NASH. However, recent studies suggested that PTX could not be efficient for the treatment of alcoholic hepatitis [164,165]. Regarding NASH, although some studies reported a beneficial effect of PTX treatment [166-168], others did not show any efficacy of this drug compared to placebo [169,170]. Moreover, steatosis, inflammation and fibrosis worsened in a few patients treated with PTX, although it was unclear whether this was related to the treatment or other factors [166,167].

Investigations carried out in wild-type (i.e. lean) mice and obese and diabetic ob/ob mice treated for 3 weeks with PTX showed that this drug aggravated fatty liver in ob/ob mice, whereas liver lipid accumulation was not observed in PTX-treated lean mice [171]. In addition, PTX further enhanced plasma ALT activity in obese mice, thus reflecting aggravation of hepatic cytolysis [171]. Another study showed that a 2-week treatment with PTX significantly increased hepatic TAG levels in mice fed a MCD diet [172]. Investigations in ob/ob mice also suggested that PTX could promote DNL in liver through a ChREBP-dependent pathway, possibly activated by PTX-induced hyperglycemia [171]. Moreover, additional investigations suggested that this hyperglycemia could be secondary to higher intestinal glucose absorption due to increased jejunal expression of the glucose transporter GLUT2 [171]. Interestingly, the methylxanthine caffeine is suspected to exaggerate post-prandial hyperglycemia in patients with T2D [173,174]. Altogether, these data suggest that long-term treatment with PTX might aggravate NAFLD in some patients, in particular in those suffering from T2D, by increasing post-prandial glycemia and hepatic DNL [171].

#### 3.2.7 Phenobarbital

Phenobarbital is a barbiturate derivative used for the treatment of epilepsy. Notably, phenobarbital is a potent inducer of different hepatic CYPs including CYP2B, CYP2C and CYP3A, in particular by activating the nuclear constitutive androstane receptor (CAR) [175,176]. Some studies also showed that phenobarbital is able to enhance CYP2E1 activity [177-179], but the mechanism of such induction is still unknown. Experimental investigations showed that phenobarbital exacerbated hepatic lipid deposition in HFD obese rats, whereas this antiepileptic drug did not induce lipid accumulation in lean rats [180]. Although the mechanism whereby phenobarbital aggravated hepatic steatosis was not determined in this study, some data might suggest a role of CYP2E1 induction. Indeed, investigations in different murine models support the notion that CYP2E1 favors hepatic lipogenesis and lipid deposition [23,181-184]. Thus, phenobarbital-induced aggravation of fatty liver in HFD obese rats [180] might have been induced, at least in part, by the exacerbation of NAFLD-related CYP2E1 induction. Alternatively, phenobarbital might have exacerbated fatty liver by activating CAR [6]. However, the role of CAR in drug-induced steatosis is still unclear and could depend on the duration of CAR activation and/or the nature of the activating molecule [6].

#### 3.2.8 Rosiglitazone

This thiazolidinedione (TZD) derivative is a PPARγ agonist used for the treatment of T2D. Indeed, TZDs enhance insulin sensitivity by different mechanisms including reduction of circulating free fatty acids, increased adiponectin secretion and stimulation of FAO and energy expenditure [185,186]. Rosiglitazone belongs to the same pharmacological class as troglitazone, which has been withdrawn from the market in 2000 because of the occurrence of several cases of severe (sometimes fatal) liver injury [187,188]. Importantly, several studies suggested that mitochondrial dysfunction could play a major role in troglitazone-induced hepatotoxicity [189,190].

Although rosiglitazone is safer than troglitazone, several cases of hepatotoxicity, sometimes severe, were reported in treated patients [31,191,192]. Interestingly, rosiglitazone also induces mitochondrial dysfunction in particular by inhibiting the activity of different MRC complexes [190,193]. However, the extent of rosiglitazone-induced inhibition of MRC complexes is less compared to troglitazone [190,193]. Pioglitazone, another antidiabetic PPARγ agonist, presents a hepatotoxicity profile and an ability to impair mitochondrial dysfunction that seems about similar to rosiglitazone [31,190]. Besides hepatotoxicity, a major concern with the use of rosiglitazone is its possible association with increased risk of cardiac diseases including congestive heart failure [186]. Rosiglitazone-induced cardiotoxicity could also involve mitochondrial dysfunction [186].

Because of their beneficial effects on insulin sensitivity, rosiglitazone and pioglitazone have been tested in the treatment of NASH [186,194]. Although pioglitazone showed beneficial effects on NASH progression, rosiglitazone efficiency was less consistent, especially during long-term treatment [194-196]. In addition, worsening of necroinflammation, perisinusoidal fibrosis and steatosis was observed in some patients [195,197]. In another study, rosiglitazone treatment was interrupted in an obese woman with severe steatosis because of a significant rise in serum ALT and AST [198]. Interestingly, increased hepatic expression of different pro-inflammatory genes (i.e. TLR4, IL-8 and CCL2) was reported in patients treated with rosiglitazone [199].

Several experimental investigations carried out in different murine models of obesity and T2D consistently reported that rosiglitazone was able to exacerbate hepatic steatosis and liver TAG accumulation [200-205]. Interestingly, two of these studies reported that aggravation of steatosis was associated with increased circulating ALT levels, thus suggesting that necroinflammation could also be worsened by rosiglitazone [200,202]. Although the exact mechanism(s) whereby rosiglitazone is able to aggravate liver steatosis in obese and diabetic mice is still unknown, some data suggest an exacerbation of hepatic DNL [200], possibly mediated by the high expression of PPARγ already present in the obese liver [202,205]. Further impairment of MRC complex I activity and exacerbation of oxidative stress might have contributed to the possible worsening of hepatic necroinflammation in rosiglitazone-treated obese mice [202].

#### 3.2.9 Tamoxifen

Tamoxifen is a selective estrogen receptor modulator used for the treatment of estrogen receptor positive breast cancer. Tamoxifen can induce different types of chronic liver lesion including steatosis, steatohepatitis, fibrosis, cirrhosis and hepatocellular carcinoma [31,78,83,206]. Whereas steatosis can be detected in at least one third of the treated patients [207,208], tamoxifen-induced steatohepatitis and cirrhosis seems to be uncommon [209-211]. Similarly, tamoxifen-induced acute hepatitis seems to be rare [31].

Several studies reported that obesity increased the risk of tamoxifen hepatotoxicity as assessed by increased plasma transaminases, or the presence of hepatic histologic alterations [211-213]. Two of these studies showed that obesity more specifically enhanced the risk of tamoxifen-induced steatohepatitis [211,213]. Interestingly, in a study reporting three cases of tamoxifen-induced steatohepatitis, all patients were over-weight, or obese [214]. However, it is noteworthy that one of the above-cited study suggested that tamoxifen did not worsen pre-existing NASH in a small subgroup of patients [211]. Hence, further investigations in larger series of obese patients undergoing serial liver biopsies would be needed in order to confirm these data. This could be clinically relevant because a previous study reported a case of NASH aggravation in a female patient treated with raloxifene [215], a selective estrogen receptor modulator used to treat osteoporosis.

The mechanism whereby tamoxifen could induce steato-hepatitis more frequently in obese individuals is currently unknown, although mitochondrial dysfunction could be involved (Figure 3). Indeed, tamoxifen is able to impair MRC activity in isolated rodent liver mitochondria *via* a direct inhibition of several MRC complexes [104,216-218]. One of these studies also showed that chronic tamoxifen treatment in mice induced mtDNA depletion and impairment of different MRC complexes in liver [216]. Because mtDNA encodes 13 MRC polypeptides [143], mtDNA depletion could also contribute to the impairment of MRC activity observed in mice treated by tamoxifen, in addition to its direct inhibitory effect on the MRC [216]. Interestingly, studies carried out in rodents showed that tamoxifen induced oxidative stress in liver, including at the mitochondrial level [219-221]. However, a single dose of tamoxifen was administered in these studies and further investigations with chronic tamoxifen treatment are required.

As previously mentioned, numerous investigations pointed to the occurrence of mitochondrial dysfunction in NAFLD. In particular, lower mitochondrial respiration with MRC substrates and reduced activity of MRC complexes have consistently been found in different rodent models of NAFLD, including in rodents with simple fatty liver or mild NASH [9,222]. Hence, it is tempting to speculate that tamoxifen might exacerbate MRC impairment in obese patients and trigger steatohepatitis in individuals with the more severe mitochondrial dysfunction and the highest ROS overproduction.

#### 3.2.10 Tetracycline

Tetracycline is a broad-spectrum antibiotic prescribed to treat different bacterial infections. However, the clinical use of tetracycline declined because the high doses required to treat infections induced numerous adverse effects. In particular, tetracycline has been responsible for several severe cases of hepatotoxicity characterized by microvesicular steatosis and fulminant liver failure [143,223]. Experimental investigations suggested that tetracycline-induced inhibition of mitochondrial FAO could be an important mechanism leading to hepatic steatosis [224-226]. A recent study suggested that one mechanism of tetracycline-induced impairment of mitochondrial FAO could be secondary to the oxidative inhibition of long-chain acyl-CoA dehydrogenase, a key enzyme of the mitochondrial FAO pathway [227]. Other investigations showed that tetracycline was able to inhibit mitochondrial protein synthesis [228-230]. The latter effect could worsen mitochondrial dysfunction during long-term tetracycline treatment because all the mtDNA-encoded proteins are mandatory for MRC activity and OXPHOS [143], as previously mentioned.

A previous study carried out in mice showed that tetracycline aggravated NAFLD in HFD obese mice [231]. Interestingly, tetracycline not only exacerbated hepatic TAG deposition but also induced steatohepatitis in obese mice, whereas no sign of tetracycline-induced hepatotoxicity was observed in lean mice [231]. Liver expression of TNFα and IL1β (two pro-inflammatory cytokines) and that of α-smooth muscle actin (α-SMA, a marker of fibrosis) were enhanced only in obese mice treated by tetracycline [231]. Unfortunately, the mechanism whereby tetracycline worsened NAFLD in obese mice was not assessed in this study. Further investigations will be needed in order to determine whether tetracycline-induced mitochondrial dysfunction and oxidative stress could be involved. It will also be interesting to assess the hepatic effects of other tetracycline derivatives in obese animals. Indeed, derivatives such as doxycycline, minocycline and rolitetracycline are also able to induce mitochondrial dysfunction [230,232,233]. Finally, it is noteworthy that tetracycline derivatives present other metabolic effects that could promote hepatic lipid accumulation including impairment of TAG secretion and stimulation of DNL [232,234,235].

## 4. General discussion and remaining issues

After reading this review, the reader might have the feeling that most of the data regarding DILI in obesity and NAFLD do not rely on solid evidence. This is right because this medical issue has been identified relatively recently and thus, only a few data have been collected thanks to a handful of clinical and experimental investigations. The sad news is that one cannot expect to have a clear picture of the situation in the next few years because there are more than 350 potential hepatotoxic drugs currently on the market [1,2], without mentioning the numerous herbals and dietary supplements that can also induce liver injury [236-238]. Importantly, many drugs, herbals and dietary supplements are taken by obese patients in order to treat different associated disorders (hyperlipidemia, T2D, hypertension), or to induce weight loss. Clearly, more investigations are needed in order to determine what are the drugs (or other compounds) that can induce liver injury in obese patients and also to decipher the involved mechanisms.

The issue of DILI in obesity is also complicated by the fact that an aggravation of NAFLD secondary to a treatment could be clinically silent in some obese individuals. Indeed, normal levels of plasma ALT and AST can be observed in a significant number of patients with NAFLD, even in those with NASH and hepatic fibrosis [239,240]. Moreover, when NAFLD is associated with increased transaminase levels, such elevation is in general modest and cannot discriminate simple steatosis from NASH [241,242]. Thus, caregivers should keep in mind that drug-induced NAFLD aggravation will not necessarily be associated with a worsening of liver enzymes and that other investigations could be needed to monitor hepatic function and histology.

Two different strategies can be implemented to collect further information regarding DILI in obesity and NAFLD. Notably, these strategies are not mutually exclusive.

First, prospective clinical studies might be designed but one should keep in mind that DILI is a rare event. Indeed, it is estimated that this adverse effect occurs at the most in about 1 per 10,000 individuals who take the drug [237,243]. Thus, it will be difficult for some drugs to draw valid conclusion because the very low number of DILI cases will preclude to statistically ascertain that obesity and/or NAFLD actually increases the risk of hepatotoxicity. Furthermore, as previously mentioned, numerous drugs are potentially hepatotoxic and thus it is difficult to *a priori* select one particular drug (or a drug class) that will be specifically studied in patients. Despite these caveats, it would be interesting to carry out prospective studies with drugs with high toxicity potential (e.g. methotrexate, tamoxifen, NRTIs) in order to confirm or not their liability in obese patients.

A second strategy is to perform experimental investigations in order to identify the drugs that might be more hepatotoxic in obesity and NAFLD. Although studies in obese rodents can be informative [53,54,121,171,202,204,231], such in vivo investigations are not amenable for the screening of a large number of drugs. In contrast, in vitro investigations appear to be the best strategy to rapidly screen a large number of drugs. In the past ten years, numerous cellular models of NAFLD have been developed by researchers willing to study the different patho-physiological consequences related to hepatocellular fat accumulation, including XME expression and drug-induced cytotoxicity [244-252]. However, most of these studies used human hepatoma cell lines (e.g. HuH7, HepG2) that do not have the full repertoire of XMEs, or rodent hepatocytes that do not have the same profile of drug metabolism than human hepatocytes. Moreover, all these studies were performed in cells incubated with fatty acids for a short duration of time (from a few hours to 2 or 3 days). This is probably too short to induce most of the hepatocellular alterations or adaptations secondary to lipid accumulation. Recently, we set up a cellular model of NAFLD by using HepaRG cells incubated with stearic acid for 7 days [24]. Importantly, CYP2E1 activity was increased whereas CYP3A4 activity was decreased in these stearate-loaded cells [24], thus reproducing what has been consistently observed in obese patients with NAFLD [8,18,25,26]. In addition, our cellular model was successfully used to demonstrate that increased APAP cytotoxicity in stearate-loaded cells was due to higher CYP2E1 activity [24]. Hence, we believe that such cellular model can be valuable to screen a great number of potentially hepatotoxic drugs. For drugs presenting significantly higher cytotoxicity in steatotic cells compared to non-steatotic cells, further studies in obese rodents might be warranted before considering clinical investigations. Even if drug metabolism presents significant differences between rodent and human, such investigations could be interesting in order to determine whether the in vitro effects can be reproduced in an in vivo situation.

Experimental investigations also offer the possibility to perform mechanistic investigations. By using rodent and cellular models of NAFLD, it will be for instance interesting to determine whether mitochondrial dysfunction is a central mechanism involved in drug-induced aggravation of NAFLD (Figure 3). Notably, almost all drugs that have been shown or suspected to worsen NAFLD are able to impair mitochondrial function, although the exact mechanisms of such mitochondrial dysfunction greatly differ from one drug to another. As already mentioned, drug-induced mitochondrial dysfunction might explain not only why hepatic fat can further accumulate but also why necroinflammation and fibrosis can be worsened in some patients. However, although numerous drugs are known to be mitochondriotoxic [4,6,104,253-255], it seems unlikely that NAFLD aggravation could be observed with all these drugs. Thus, additional mechanisms unrelated to mitochondrial dysfunction might also be involved such as induction of ER stress, JNK activation, impairment of autophagy and the accumulation of certain lipid intermediates since these events could play a significant role in NAFLD progression [256-258]. Finally, it would be also interesting to determine whether the drugs shown or suspected to worsen NAFLD are able to reduce the expression and activity of patatin-like phospholipase domain containing 3 (PNPLA3), a lipase which appears to play a major role in the occurrence of fatty liver and its progression to steatohepatitis [259,260].

## ABBREVIATIONS

ALF: acute liver failure;
ALI: acute liver injury;
ALT: alanine aminotransferase;
APAP: acetaminophen;
AST: aspartate aminotransferase;
AZT: zidovudine;
BMI: body mass index;
CAR: constitutive androstane receptor;
ChREBP: carbohydrate responsive element-binding protein;
CYP: cytochrome P450;
ddI: didanosine;
DILI: drug-induced liver injury;
DNL: de novo lipogenesis;
d4T: stavudine;
ER: endoplasmic reticulum;
FAO: fatty acid oxidation;
GSH: glutathione;
HFD: high-fat diet;
IR: insulin resistance;
MCD: methionine choline-deficient;
MRC: mitochondrial respiratory chain;
MTX: methotrexate;
mtDNA: mitochondrial DNA;
NAFLD: nonalcoholic fatty liver disease;
NAPQI: N-acetyl-p-benzoquinone imine;
NASH: nonalcoholic steatohepatitis;
NEFA: non-esterified fatty acids;
NRTI: nucleoside reverse transcriptase inhibitor;
OXPHOS: oxidative phosphorylation;
PPAR: peroxisome proliferator-activated receptor;
PTX: pentoxifylline;
ROS: reactive oxygen species;
SREBP-1c: sterol regulatory element-binding protein-1c;
TAG: triacylglycerol;
TCA: tricarboxylic acid;
T2D: type 2 diabetes;
TNFα: tumor necrosis factor-α;
TZD: thiazolidinedione;
VLDL: very low-density lipoprotein;
XME: xenobiotic-metabolizing enzyme;
WAT: white adipose tissue.
